# Association among biomarkers, phenotypes, and motor milestones in Chinese patients with 5q spinal muscular atrophy types 1–3

**DOI:** 10.3389/fneur.2024.1382410

**Published:** 2024-09-02

**Authors:** Shijia Ouyang, Xiaoyin Peng, Wenchen Huang, Jinli Bai, Hong Wang, Yuwei Jin, Hui Jiao, Maoti Wei, Xiushan Ge, Fang Song, Yujin Qu

**Affiliations:** ^1^Department of Medical Genetics, Capital Institute of Pediatrics, Beijing, China; ^2^Department of Neurology, Children's Hospital Affiliated to Capital Institute Pediatrics, Beijing, China; ^3^Center of Clinical Epidemiology, TEDA International Cardiovascular Hospital, Tianjin, China

**Keywords:** spinal muscular atrophy, biomarkers, severity, motor milestones, survival

## Abstract

**Background:**

Biomarkers can be used to assess the severity of spinal muscular atrophy (5q SMA; SMA). Despite their potential, the relationship between biomarkers and clinical outcomes in SMA remains underexplored. This study aimed to assess the association among biomarkers, phenotypes, and motor milestones in Chinese patients diagnosed with SMA.

**Methods:**

We collected retrospective clinical and follow-up data of disease-modifying therapy (DMT)-naïve patients with SMA at our center from 2019 to 2021. Four biomarkers were included: survival motor neuron 2 (SMN2) copies, neuronal apoptosis inhibitory protein (NAIP) copies, full-length SMN2 (*fl*-SMN2), and F-actin bundling protein plastin 3 (PLS3) transcript levels. Data were analyzed and stratified according to SMA subtype.

**Results:**

Of the 123 patients, 30 were diagnosed with Type 1 (24.3%), 56 with Type 2 (45.5%), and 37 with Type 3 (30.1%). The mortality rate for Type 1 was 50%, with median survival times of 2 and 8 months for types 1a and 1b, respectively. All four biomarkers were correlated with disease severity. Notably, *fl*-SMN2 transcript levels increased with SMN2 copies and were higher in Type 2b than those in Type 2a (*p* = 0.028). Motor milestone deterioration was correlated with SMN2 copies, NAIP copies, and *fl*-SMN2 levels, while PLS3 levels were correlated with standing and walking function.

**Discussion:**

Our findings suggest that SMN2 copies contribute to survival and that *fl*-SMN2 may serve as a valuable biomarker for phenotypic variability in SMA Type 2 subtypes. These insights can guide future research and clinical management of SMA.

## Background

5q Spinal Muscular Atrophy (5q SMA) is a hereditary neuromuscular disease with a high incidence and mortality rate. It is an autosomal recessive genetic disease caused by progressive degeneration and loss of α-motor neurons, which manifests clinically as progressive muscular weakness and atrophy. 5q SMA (hereafter referred to simply as “SMA”) is caused by a homozygous deletion or compound heterozygous variation of the motor neuron survival gene 1 (SMN1), which is located at 5q13.2 (1). The incidence of SMA is approximately 1/10,000 (2) and the carrier rate in the Chinese population is approximately 1/48–1/42 (3).

The disease manifests as a broad spectrum of clinical symptoms (4). In accordance with the consensus diagnosis of SMA, patients are categorized into one of five types (Type 0 to Type 4) based on the age at onset and motor milestones achieved. Patients with SMA Type 1 are classified into three subtypes (1a, 1b, and 1c) based on their head control ability and age at symptom onset. Patients with SMA Type 2 are further classified into subtypes 2a and 2b based on their age at onset and ability to maintain an independent sitting function. Patients with SMA Type 3 are subdivided into subtypes 3a and 3b based on their age at onset (4, 5).

With notable advancements in SMA treatment, the Food and Drug Administration has approved three therapies: nusinersen, risdiplam, and onasemnogene abeparvovec. Although most patients are with SMN1 homozygous deletion, significant differences in disease progression, prognosis, and survival have been observed among patients with different phenotypes (4). Furthermore, there is considerable uncertainty in response to intervention, treatment efficacy, and long-term outcomes (6). Biomarkers offer valuable means of evaluating disease progression, prognosis, and response to therapy. It is imperative to ascertain the correlation between responsive outcome measures and the spectrum of disease severity for individual patient follow-up. Numerous SMA biomarkers have been investigated globally, including the survival motor neuron 2 (SMN2) copy number, SMN messenger RNA (mRNA) and protein levels, neurofilament proteins, creatine kinase, creatinine, and a range of electrophysiological and imaging measures (6, 7). However, a single biomarker may not provide a comprehensive interpretation of phenotypic heterogeneity. Therefore, studies that include multiple biomarkers may provide novel and valuable clarifications.

Although the clinical characteristic data (including endpoint survival and motor function) of subtypes have been studied in disease-modifying therapy (DMT) treatment-naïve patients with SMA (8, 9), little data are available for Chinese patients with SMA. With the approval of the modified drug (nusinersen) by the relevant medical insurance authorities in China in December 2021, an increasing number of patients are undergoing treatment. Therefore, it is imperative to have a comprehensive understanding of the baseline data on the clinical characteristics and biomarkers of DMT treatment-naïve patients to assess the long-term treatment effects. The objective of this study was to investigate the endpoint survival, phenotypic severity, motor milestone deterioration, and four biomarkers [SMN2 and neuronal apoptosis inhibitory protein (NAIP) copy numbers, and transcript levels of full-length SMN2 (fl-SMN2) and F-actin bundling protein plastin 3 (PLS3)] in Chinese patients with SMA types 1, 2, and 3, and their subtypes. In addition, we sought to ascertain whether SMA biomarkers were correlated with disease severity and responded to motor function deterioration.

## Methods

### Design and participants

This follow-up study included 123 patients diagnosed with SMA Type 1–3 and SMN1 gene homozygous deletion. The patients were registered in the National Rare Disease Registry System between January 2019 and December 2021 (10, 11). Patients with compound heterozygous variations in the SMN1 gene or those who had received DMT treatment were excluded from the study. Patient enrollment commenced on January 1, 2019, and baseline data and retrospective medical records were collected as previously reported (10). Subsequently, prospective follow-up was conducted until December 2021.

A total of 123 unrelated patients with homozygous deletion of SMN1 were diagnosed with SMA by genetic testing in our laboratory. Information regarding patients’ clinical records and survival status was obtained from a questionnaire designed by a multidisciplinary team comprising geneticists, neurologists, and medical statisticians. The dataset included information on age at onset, sex, family history, motor milestones, disease progression, survival status, and treatment or care measures, as previously described in our studies (10, 12, 13). Follow-up information was collected by telephone, WeChat, and email. To ensure consistency and accuracy between the input data and original information, the questionnaires obtained via WeChat or email were cross-checked and entered into the database. In addition, the follow-up questionnaire included retrospective data, such as the age of onset and achievement of motor milestones. In the event of inconsistencies between this follow-up and the previous follow-up, the answer was verified based on the medical report. In addition, spinal radiographic reports were gathered to evaluate spinal deformities in patients with suspected scoliosis. The onset age was defined as when the first abnormalities appeared in records or were reported by parents. The conventional composite endpoints for survival were death and mechanical ventilation for ≥16 h/d.

### Classification criteria

Patients were divided into three categories based on their age at onset and the motor milestones they had acquired by the end of the follow-up period. The first category, Type 1, included patients who had never acquired the ability to sit without support. The second category, Type 2, included patients who could sit without support but could not stand or walk without assistance. The third category, Type 3, included patients who could walk independently. The criteria for sub-classification are summarized in Table 1, as previously published (4, 5, 9, 14). In the event of discrepancies between the age at onset and the highest achieved motor milestone, the final classification was determined by the maximum motor function measured until the end of follow-up. During the follow-up period, individual patient subtypes were adjusted based on the progress observed in motor milestones. Of the 123 patients, 30 were classified as Type 1, 56 as Type 2, and 37 as Type 3. The latter group comprised 59 males and 64 females.

**Table 1:** Classification and subtypes of SMA types 0–4.

SMA type	Subtype	Age at onset	Sports milestones and complications
Type 0		Prenatal	Never achieves head control
Type 1		0–6 months	Never sits
	1a	<1 months	Head control absent, feeding difficulty, need for respiratory support
	1b	<3 months	Poor or absent head control
	1c	>3 months	Head control and rolling achieved
Type 2		6–18 months	Sits but never walks
	2a	6 ~ 12 months	Sits independently, may lose the ability to sit later in life
	2b	>12 months	Sits independently, maintains the ability to sit for a long time
Type 3		>18 months	Stands and walks
	3a	18 months–3 years	Usually early loss of ambulation
	3b	>3 years	Usually preserved walking until adulthood
Type 4		>20 years	Preserved walking ability

SMA, spinal muscular atrophy.

### Copy number detection of the SMN and NAIP genes

Genomic DNA was extracted from peripheral blood using a DNA mini kit (QIAGEN, Beijing, China), and SMN1, SMN2, and NAIP copy numbers were determined via multiplex ligation-dependent probe amplification (MLPA) analysis using the SALSA MLPA kit P021-B1-01 (MRC-Holland, Amsterdam, The Netherlands) according to the manufacturer’s instructions (15).

### Detection of fl-SMN2 and PLS3 transcript levels

A total of 81 patients consented to the transcript-level analysis by their guardians. Total RNA was extracted from the peripheral blood using an RNA Simple Total RNA Kit (Tiangen, Beijing, China). A total of 800 ng RNA was subjected to reverse transcription for 60 min at 37°C. This was achieved through the use of 3.75 mM random primers (Sangon, Shanghai, China) and 2 μL of M-MLV reverse transcriptase (Invitrogen, Carlsbad, CA, United States), with a total volume of 40 μL. Real-time PCR was performed to assess the expression levels of fl-SMN2 and PLS3. The *fl*-SMN2 mRNA levels were normalized to those of GAPDH. Absolute real-time PCR was conducted to quantify fl-SMN2 transcripts using a 7500 real-time PCR system (ABI Q7, Applied Biosystems, United States). The thermal cycling conditions were as follows: 50°C for 2 min, 95°C for 10 min, 40 cycles at 95°C for 15 s, and 60°C for 1 min (16–18). Primers and minor groove binder (MGB) probes were synthesized by Invitrogen. The primers and MGB probe sequences for glyceraldehyde 3-phosphate dehydrogenase (GAPDH) were as follows: forward primer sequence, 5′-GGGTGTGAACCATGAGAAGTATGA-3′; reverse primer sequence, 5′-CTAAGCAGTTGGTGGTGCAGG-3′; and fluorescent reporter sequence, 5′-FAM-CAAGATCATCAGCAATGC-NFQ. For SMN2, the primers and MGB probe sequences were as follows: 5′-TGGTACATGAGTGGCTATCATA CTG-3′, 5′-GTGAGCACCTTCCTTCTTTTT-3′, and 5′-FAM-ATGGGTTTTAGAA-MGB-NFQ. Relative quantification real-time (RT)-PCR was used to quantify the PLS3 transcript levels. The PLS3 cDNA was amplified using the primer pair 5′-TGGCTACCACTCAGATTTCCAA-3′ and 5′-GAATCCGTTGCTGTTGAGATCA-3′. The primer sequences for GAPDH were identical to those previously described. The 20 μL RT-PCR reaction mixture included 1 × SYBR Green PCR mixture (Kangwei, Beijing, China), 5 μL of PLS3 cDNA, and 0.25 pmol μL^−1^ of each primer. The RT-PCR conditions were as follows: an initial denaturation step at 95°C for 1 min, followed by 40 cycles at 95°C for 15 s, and 64°C for 1 min. PLS3 mRNA levels were normalized to GAPDH mRNA levels.

### Statistical analysis

Quantitative data are presented as mean ± standard deviation or median with minimum and maximum values. The Student’s *t*-test or one-way ANOVA was employed for comparisons between groups, whereas the Student–Newman–Keuls test was used for multiple comparisons among groups. Kendall’s tau-c test was used to evaluate the correlation between biomarkers and their types, subtypes, and motor milestones. Qualitative data were described as percentages, and comparisons between groups were performed using the chi-square test, corrected chi-square test, or Fisher’s exact test. Endpoint survival analysis and motor milestone deterioration were conducted using the Kaplan–Meier method, whereas the log-rank test was employed to compare differences between SMA types, subtypes, and groups. Endpoint survival analyses were conducted using logistic or Cox models to accommodate the multifactorial nature of data. A backward elimination method was employed in the variable screening process. The analysis and processing of research data were conducted by professional statisticians using the SPSS software, version 26.00 (IBM Corp., Armonk, NY). The probability of the variables entering the equation was set at 0.05, whereas the exclusion was set at 0.10. All other parameters were set to default values. All tests were two-sided, and *p <* 0.05 was considered statistically significant.

## Results

### Clinical characteristics of 123 patients in our cohort

The clinical characteristics of the cohort are presented in Supplementary Table S1. Of the 123 patients, 24.3% were diagnosed with Type 1, 45.5% with Type 2, and 30.1% with Type 3. No significant differences were observed in sex distribution among the three types (*p >* 0.05). However, significant differences were noted in the median age, median age at onset, median age at feeding difficulties, and median age at diagnosis of scoliosis among types 1–3 (*p <* 0.001, *p <* 0.001, *p <* 0.001, *p =* 0.012, and *p <* 0.001, respectively). The median ages at the onset of SMA types 1–3 were 3, 10, and 21 months, respectively. Fifteen patients with SMA Type 1 (50%) met the conventional composite endpoints, including 14 deaths and 1 patient who required permanent ventilation. In contrast, no composite endpoints were observed in patients with SMA types 2 or 3. In patients with SMA Type 1, the rate of respiratory support was 10%. Although 36.7% of patients with SMA Type 1 had a history of feeding difficulties, none of the patients in the cohort required gastrostomy tube feeding. The results of the motor milestones demonstrated that 46.7% of patients with SMA Type 1 achieved head control, whereas 23.3, 91.1, and 100% of patients with SMA types 1–3, respectively, could roll over. In addition, 8.9% of the patients with SMA Type 2 could stand. In contrast, patients with SMA Type 3 demonstrated full attainment of all the motor milestones.

### Clinical characteristics of SMA type 1 subtypes

**Figure 1:**
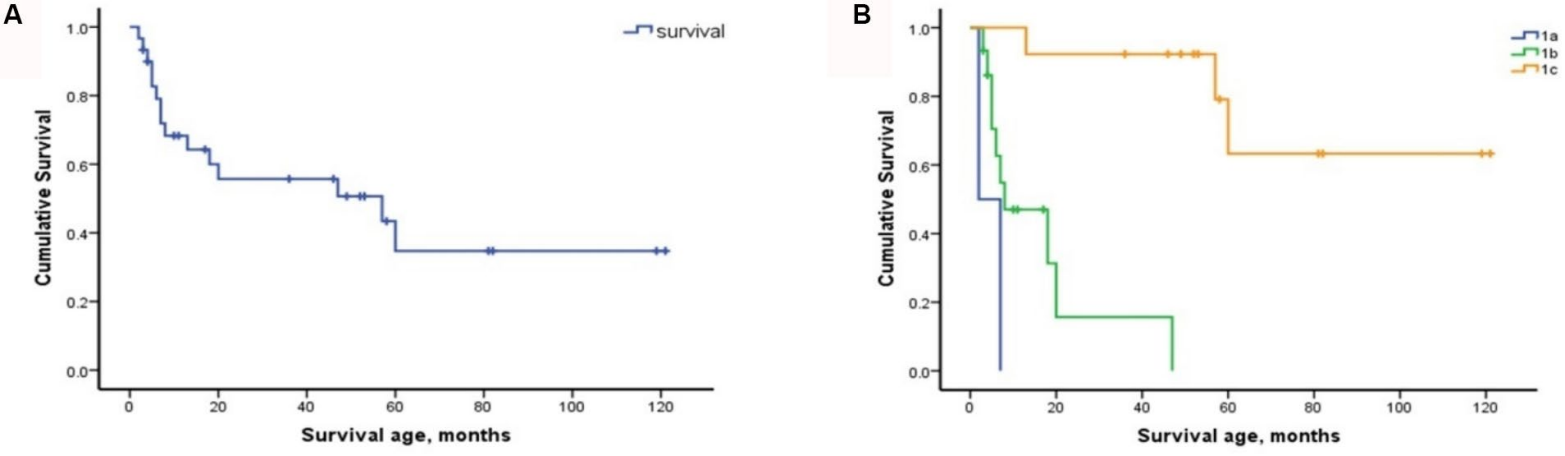
Composite endpoint survival curves of patients with SMA Type 1 and subtypes. **(A)** Composite endpoint survival curve of patients with SMA Type 1 disease using Kaplan–Meier analysis. **(B)** Composite endpoint survival curve of patients with SMA types 1a, 1b, and 1c using Kaplan–Meier analysis. SMA, spinal muscular atrophy; NAIP, neuronal apoptosis inhibitory protein.

The cohort comprised 30 patients with SMA Type 1, comprising two patients with Type 1a, 15 with Type 1b, and 13 with Type 1c. The clinical characteristics of the patients are presented in Table 2. The mortality rates of SMA types 1a, 1b, and 1c were 100, 66.7, and 23.1%, respectively. Kaplan–Meier survival analysis revealed a correlation between age at onset and the probability of endpoint-free survival in patients with SMA Type 1 (Figure 1A). Furthermore, the probability of endpoint-free survival exhibited notable variation among patients with the three subtypes of Type 1 SMA (*χ*^2^ = 23.209, *p <* 0.001) (Figure 1B). Patients with SMA Type 1a exhibited the most severe phenotype and shortest survival age, with a median survival age of 2 months. The phenotype remained severe in patients with SMA Type 1b, with a median survival of 8 months. The data on endpoint-free survival probability indicated that patients with SMA Type 1c exhibited a superior survival status compared to those with SMA types 1a and 1b (Table 3).

**Table 2:** Clinical characteristics of SMA types 1a, 1b, and 1c.

	Type 1a (*n* = 2)	Type 1b (*n* = 15)	Type 1c (*n* = 13)	Statistic	*p-*value
Sex (M: F)	0:2	2:13	9:4	Fisher’s exact test	**0.001**
Age, months^a^	4.5 (2–7)	7 (3–47)	57 (13–121)	19.684	**<0.0001**
Age at onset, months^a^	0.6 (0.4–0.7)	1.5 (1–3)	5 (1–8)	9.795	**0.001**
Endpoint, *n* (%)	2 (100%)	10 (66.7%)	3 (23.1%)	Fisher’s exact test	**0.036**
Median survival age, months^a^	2	8	–		
History of feeding difficulties, *n* (%)	1 (50)	6 (40)	4 (30.8)	Fisher’s exact test	1.000
Motor milestones, *n* (%)					
Head control	0 (0)	1 (6.7)	13 (100)	Fisher’s exact test	**<0.001**
Head control deterioration	–	1 (100)	4 (30.8)		
Rolling	0 (0)	0 (0)	7 (53.8)	Fisher’s exact test	**0.0014**
Rolling deterioration	–	–	6 (85.7)		

^a^Median (minimum-maximum). F, female; M, male; NA, not applicable; SMN, survival motor neuron; SMA, spinal muscular atrophy; NAIP, neuronal apoptosis inhibitory protein; PLS3, F-actin bundling protein plastin 3. Bold in the table indicates *p* < 0.05.

**Table 3:** Endpoint-free survival probabilities in SMA by age.

Endpoint-free survival probabilities by age, y									
SMA type		No.	1	2	4	5	10	20	60
Our study									
	1a	2	0	NA	NA	NA	NA	NA	NA
	1b	15	47.0	15.7	0	NA	NA	NA	NA
	1c	13	100	92.3	92.3	63.3	63.3	NA	NA
Wijngaarde et al. (9)									
	1a	3	0	NA	NA	NA	NA	NA	NA
	1b	35	30.6	6.5	0.2	0.03	NA	NA	NA
	1c	32	97.7	94.9	88.5	85.3	69.3	43.0	4.2

NA, not available.

### Clinical characteristics of the subtypes of SMA type 2

Type 2 patients were subdivided into Type 2a (*n* = 29) and 2b (*n* = 27) (Table 4). No significant differences were observed between the two subtypes regarding sex, age, feeding difficulties, SMN2 and NAIP copy numbers, or PLS3 expression (*p >* 0.05). Nevertheless, a significant difference was observed in the median age of onset (*p =* 0.011). The median age at onset was 8 months for SMA Type 2a and 12 months in 2b. Although the incidence of scoliosis did not differ between the two subtypes, the age at which spinal deformities occurred in SMA Type 2b was 47.5 months, which was significantly later than that in SMA Type 2a at 36 months (*t* = 2.520, *p =* 0.021).

**Table 4:** Clinical characteristics of SMA types 2a and 2b.

	Type 2a (*n* = 29)	Type 2b (*n* = 27)	Statistic	*P-*value
Sex (M:F)	14:15	15:12	0.076	0.781
Age, months^a^	58 (11–232)	62 (23–193)	*t* = −1.000	0.322
Age at onset, months^a^	8 (4–18)	12 (6–18)	*t* = −2.626	**0.011**
History of feeding difficulties, *n* (%)	3 (10.3)	2 (7.4)	*t* = 0.379	0.706
Age of feeding difficulties, months^a^	22 (14–180)	17.5 (11–24)	*t* = 0.779	0.493
Scoliosis, *n* (%)	11 (37.9)	10 (37.0)	0.005	0.945
Age at diagnosis of scoliosis, months^a^	36 (8–72)	47.5 (24–108)	*t* = −2.520	**0.021**
Motor milestones, *n* (%)				
Rolling	25 (86.2)	26 (96.3)	*χ*^2^ = 1.75	0.1858
Rolling deterioration	7 (28.0)	3 (11.5)	*χ*^2^ = 2.19	0.1388
Sitting	29 (100)	27 (100)		
Sitting deterioration	8 (27.6)	1 (3.7)	*χ*^2^ = 5.91	**0.015**
Standing	0 (0)	5 (18.5)	Fisher’s exact test	**0.0257**
Standing deterioration	–	2 (40.0)		

^a^Median (minimum-maximum). F, female; M, male; NAIP, neuronal apoptosis inhibitory protein; PLS3, F-actin bundling protein plastin 3; SMA, spinal muscular atrophy; SMN, survival motor neuron. Bold in the table indicates *p* < 0.05.

### Clinical characteristics of the SMA type 3 subtypes

A total of 28 patients with Type 3a and nine patients with Type 3b were enrolled in the study. Age, median age at onset, and age at diagnosis of scoliosis differed between the two subtypes (Table 5).

**Table 5:** Clinical characteristics of SMA types 3a and 3b.

	Type 3a (*n* = 28)	Type 3b (*n* = 9)	Statistic	*P-*value
Sex (M:F)	16:12	3:6	Fisher’s exact test	**0.001**
Age, months^a^	71 (33–288)	169 (58–518)	*t* = −3.788	**0.001**
Age at onset, months^a^	18 (10–31)	36 (36–180)	*t* = −4.696	**<0.001**
Scoliosis, *n* (%)	6 (21.4)	2 (22.2)	Fisher’s exact test	0.657
Age at diagnosis of scoliosis, months^a^	90 (28–130)	211.5 (135–288)	*t* = −2.726	**0.034**
Motor milestones, *n* (%)				
Standing	28 (100)	9 (100)		
Standing deterioration, *n* (%)	5 (17.9)	2 (22.2)	*χ*^2^ = 0.0846	0.7712
Walking	28 (100)	9 (100)		
Walking deterioration, *n* (%)	9 (32.1)	3 (33.3)	*χ*^2^ = 0.0044	0.9443

^a^Median (minimum–maximum). F, female; M, male; SMN, survival motor neuron. Bold in the table indicates *p* < 0.05.

### Motor milestone deterioration of 123 patients in our cohort

**Figure 2:**
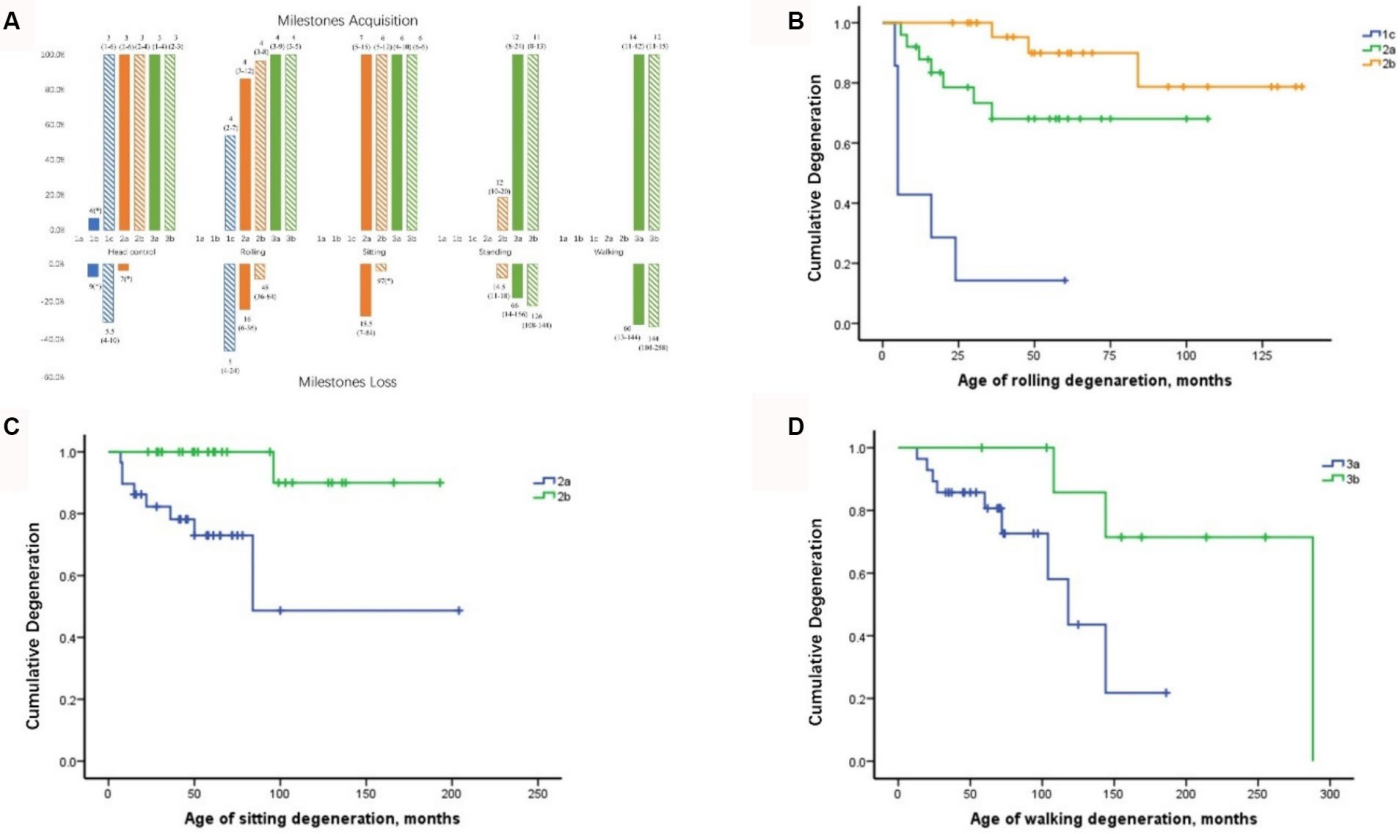
Milestone acquisition and loss in patients with different SMA subtypes and curves of motor milestone deterioration. **(A)** Milestone acquisition and loss in patients with different SMA subtypes. The number indicates the age at milestone acquisition or loss, shown as the median (minimum–maximum). The percentage in the figure represents the proportion of milestone acquisition or loss cases within subtype groups. * Indicates that only one case showed milestone acquisition or loss. SMA, spinal muscular atrophy. **(B)** Curve of rolling deterioration in SMA subtypes 1c, 2a, and 2b using Kaplan–Meier analysis. **(C)** Curve of sitting deterioration in SMA subtypes 2a and 2b using Kaplan–Meier analysis. **(D)** Curve of walking deterioration in SMA subtypes 3a and 3b using Kaplan–Meier analysis.

The acquisition and deterioration rates of motor milestones in patients with each SMA subtype are presented in Tables 2, 4, 5, respectively. A summary of motor milestone acquisition or loss status is shown in Figure 2A. The results demonstrated significant discrepancies in the acquisition or deterioration of motor milestones across the seven subtypes. The median ages of acquisition for patients with head control, rolling, sitting, standing, and walking were 3, 4, 6, 12, and 14 months, respectively. The time span of functional deterioration was calculated for each motor milestone and defined as the age of deterioration minus the age of acquisition. The shortest deterioration average span time was observed for head control (3.17 months), followed by rolling (16.53 months), sitting (29.44 months), standing (60.78 months), and walking (75.5 months).

All patients with SMA Type 1c achieved head control by an average of 3 months, with four cases demonstrating deterioration at 5.5 months. Furthermore, seven cases of Type 1c exhibited rolling ability, of which six (85.7%) subsequently deteriorated. The rolling deterioration rates for types 2a and 2b were 29.2 and 11.5%, respectively. Kaplan–Meier analysis revealed that rolling deterioration status differed significantly (*p =* 0.001) among patients with SMA types 1c, 2a, and 2b (Figure 2B). The median ages at rolling deterioration for SMA types 1c, 2a, and 2b were 5, 16, and 48 months, respectively.

Most cases of sitting deterioration occurred in patients with Type 2a (27.6%), with a median age at deterioration of 18.5 months. In the Type 2b group, only one patient exhibited independent sitting deterioration at 97 months. Kaplan–Meier sitting deterioration analysis revealed a statistically significant difference between SMA types 2a and 2b (*p =* 0.003) (Figure 2C).

Regarding standing function, the deterioration rate and the median age at deterioration exhibited no discernible differences between SMA types 3a and 3b (*p >* 0.05). The median age at which walking deterioration occurred in patients with types 3a and 3b was 60 and 144 months, respectively. Kaplan–Meier analysis revealed that the walking deterioration status of SMA Type 3a was more severe than that of SMA Type 3b (*χ*^2^ = 4.255, *p =* 0.039) (Figure 2D).

### Biomarkers of the cohort

#### Relationships between clinical phenotypes and biomarkers in SMA types 1–3

**Figure 3:**
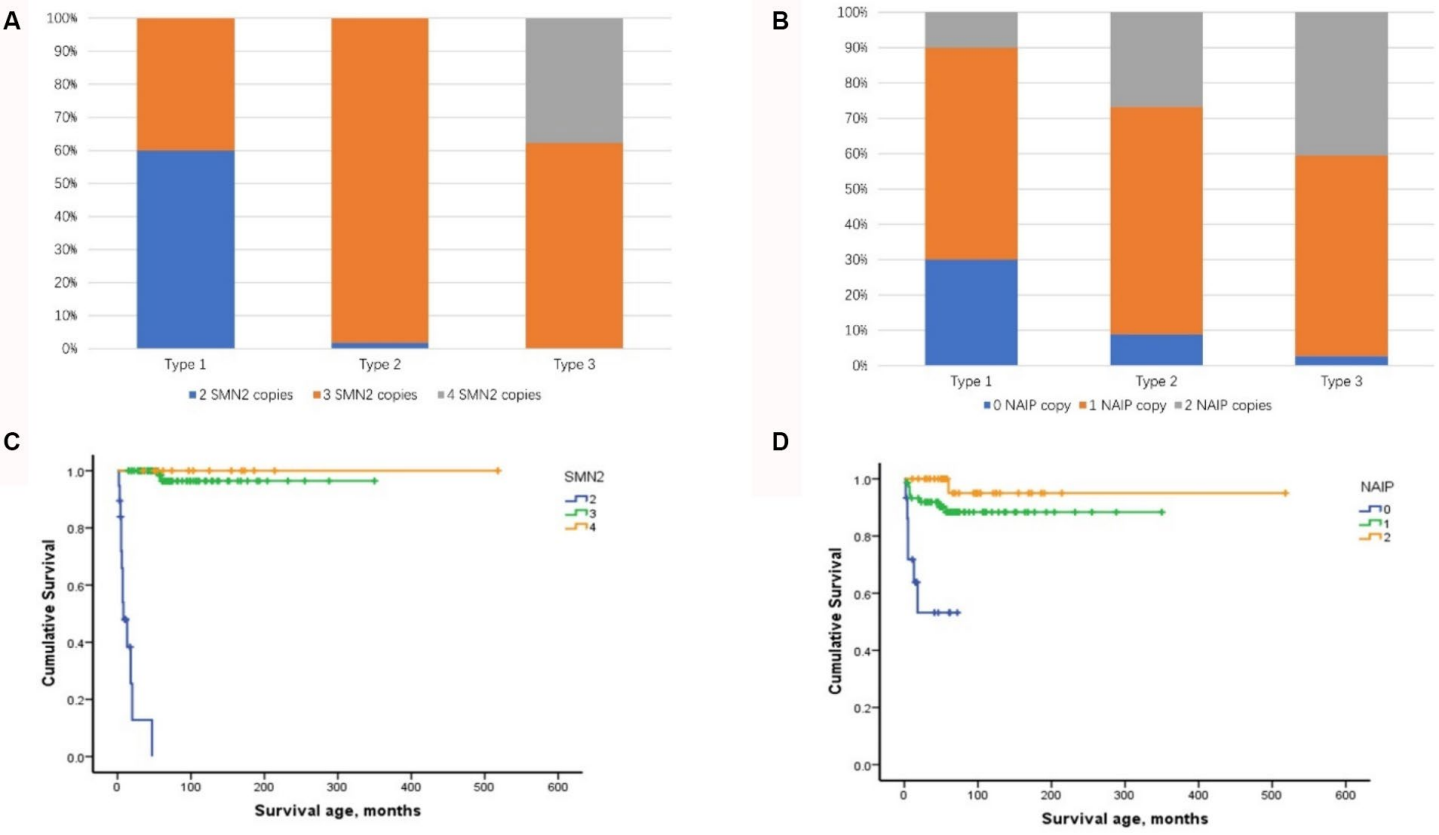
Distribution of SMN2 and NAIP copy numbers and their impact on composite endpoint survival in patients with SMA types 1–3. **(A)** Distribution of SMN2 copy numbers in patients with SMA types 1–3. **(B)** Distribution of NAIP copy numbers in patients with SMA types 1–3. **(C)** Impact of SMN2 copy number on composite endpoint survival in patients with SMA types 1–3. **(D)** Impact of NAIP copy number on composite endpoint survival in patients with SMA types 1–3. SMA, spinal muscular atrophy; NAIP, neuronal apoptosis inhibitory protein.

Among the four biomarkers, copies of the SMN2 and NAIP genes were detected in 123 patients and were negatively correlated with phenotype severity (Kendall’s tau-c = −0.519, *p* < 0.001 and Kendall’s tau-c = −0.274, *p <* 0.001, respectively) (Figures 3A,B). A significant difference was observed in the age of onset among patients with SMA with different SMN2 copies (*χ*^2^ = 57.728, *p <* 0.001) and NAIP copies (*χ*^2^ = 19.585, *p <* 0.001). The age at onset in patients with 2, 3, and 4 SMN2 copies was 1.5, 11, and 24 months, respectively, whereas the age at onset in patients with 0, 1, and 2 NAIP copies was 3, 11, and 12 months, respectively. The survival analysis data are illustrated in Figure 3C, which depicts the endpoint-free survival probabilities at 1 and 2 years in patients with two SMN2 copies, which were 47.9 and 0%, respectively. The median survival time was 7 months. In patients with three or four SMN2 copies, the endpoint-free survival probability at 1 year was 100%, whereas the endpoint-free survival probabilities at 5 years were 96.4 and 100%, respectively. Log-rank analysis revealed a statistically significant difference among the three groups (*χ*^2^ = 137.821, *p <* 0.001). Regarding the NAIP gene, the endpoint-free survival probabilities at 1 year for patients with zero, one, and two NAIP copies were 71.8, 91.9, and 100%, respectively. Furthermore, log-rank analysis revealed significant differences among the three groups (*χ*^2^ = 21.952, *p <* 0.001) (Figure 3D).

Eight combined SMN1-SMN2-NAIP genotypes (0–2-0, 0–2-1, 0–2-2, 0–3-0, 0–3-1, 0–3-2, 0–4-1, and 0–4-2) were identified in the cohort. The 0–3-1 genotype was the most prevalent, occurring in 65 cases (52.8%) and predominantly in patients with types 2 and 3. Furthermore, the distribution of the SMN1-SMN2-NAIP genotype was correlated with phenotype severity (Kendall’s tau-c = −0.475, *p <* 0.001). Patients with 0–2-0 or 0–2-1 exhibited a more severe phenotype, with an earlier age of onset and higher mortality rate.

**Figure 4:**
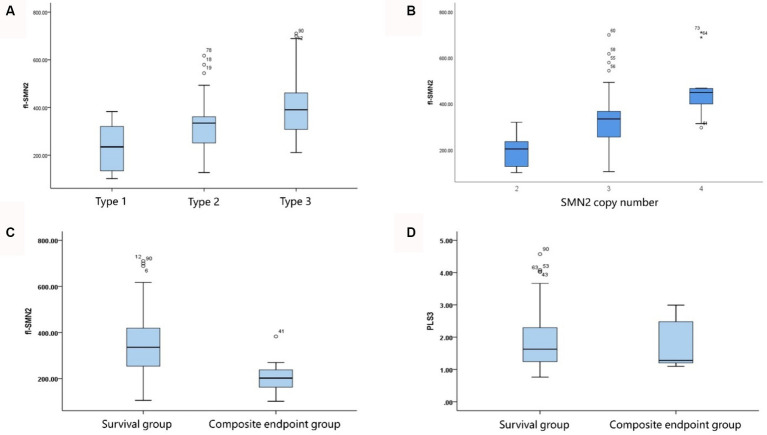
Correlation of fl-SMN2 expression with phenotype severity and SMN2 copy number. **(A)** Correlation between fl-SMN2 expression and phenotype severity. **(B)** Correlation between fl-SMN2 expression and SMN2 copy number. **(C)** *fl*-SMN2 transcript levels in the composite endpoint and survival groups of patients with Type 1–3 SMA and **(D)** PLS3 transcript levels in the composite endpoint and survival groups of patients with Type 1–3 SMA.

The analysis of transcript levels of biomarkers revealed that fl-SMN2 and PLS3 transcript levels were quantified in 81 patients and correlated with the severity of the phenotype (*F* = 21.762, *P*_trend_ < 0.001 and *F* = 5.018, *P*_trend_ = 0.028, respectively) (Supplementary Table S1 and Figure 4A). The fl-SMN2 transcript level was 196.95 ± 68.77, 331.22 ± 116.69, and 456.16 ± 122.34 in patients with two, three, and four SMN2 copies, respectively. This demonstrated an increasing trend with increasing SMN2 copy numbers (*F* = 33.350, *p <* 0.001) (Figure 4B). The fl-SMN2 transcript level in the composite endpoint group was markedly lower than that in the survival group (*F* = 6.485, *p* = 0.012) (Figure 4C). Analysis of PLS3 transcript levels revealed no significant differences between the sexes or age groups, and no discernible difference in PLS3 transcript levels between the survival and composite endpoint groups (Figure 4D). However, the results indicated a tendency for higher levels in female patients (1.98 ± 0.92) than those in male patients (1.78 ± 0.79) and for higher levels in female patients aged >36 months (2.03 ± 0.97) than in those aged <36 months (1.86 ± 0.83).

Analysis of the correlation between the biomarkers and subtype phenotypes of patients with SMA Type 1 indicated that the distribution of *SMN2* and *NAIP* copies was negatively correlated with disease severity (Kendall’s tau-c = −0.907, *p* < 0.001; Kendall’s tau-c = −0.383, *p* = 0.007, respectively). A reduction in the number of copies carried was associated with an increase in the severity of the phenotype and a reduction in the survival age. The composite endpoint survival status can be significantly improved when the number of SMN2 copies reaches three or more. In this study, fl-SMN2 and PLS3 transcript levels were not obtained in patients with SMA Type 1a because of early demise. The data for patients with types 1b and 1c are compared herein. The results demonstrated that the fl-SMN2 transcript level in SMA Type 1c was significantly higher than that in SMA Type 1b (*p* = 0.018). However, no notable difference was observed in PLS3 transcript levels between the two subtypes (*p* = 0.143) (Table 6). The expression level of fl-SMN2 in SMA Type 2b was 365.46 ± 116.00, which was significantly higher than that observed in Type 2a (*t* = 2.289, *p* = 0.028). No correlation was identified between the four biomarkers and types 3a or 3b. Nevertheless, an upward trajectory in the expression levels of fl-SMN2 and PLS3 was discerned in females with SMA Type 3b compared to those with SMA Type 3a (Supplementary Figure S1).

**Table 6:** Biomarkers data in SMA Subtypes 1–3.

		Type 1a	Type 1b	Type 1c	Type 2a	Type 2b	Type 3a	Type 3b
Numbers^*^		2	15	13	29	27	28	9
SMN2	2 copies	2	15	1	1	0	0	0
	3 copies	0	0	12	28	27	19	4
	4 copies	0	0	0	0	0	9	5
NAIP	0 copy	1	7	1	3	2	1	0
	1 copy	1	8	9	19	17	18	3
	2 copies	0	0	3	7	8	9	6
Numbers^#^		0	11	6	18	18	20	8
fl-SMN2		NA	193.41 ± 70.96	303.624 ± 99.60	281.10 ± 104.84	365.46 ± 116.00	380.12 ± 109.28	456.52 ± 170.73
PLS3		NA	1.785	1.422	1.83 ± 0.68	1.65 ± 0.64	2.09 ± 1.04	2.51 ± 1.21

Numbers^*^ indicate that 123 patients had copy numbers of SMN2 and NAIP. Numbers^#^ indicates that the guardians of 81 patients consented to undergo transcription-level analysis of fl-SMN2 and PLS3.

Cox regression analysis (forward conditional method) was performed for multivariate survival analysis in the context of SMA Type 1. fl-SMN2 and PLS3 transcript levels were not included in the Cox regression analysis because of the unavailability of RNA in a subset of patients. Among the factors considered, including age at onset, sex, feeding difficulty, respiratory support, SMN2 and NAIP copy numbers, and attainment of head control or rolling, only the SMN2 copy number was negatively correlated with the occurrence of composite endpoints in Type 1 patients (*B* = −5.113, *p* = 0.031).

#### Effects of biomarkers on motor milestone deterioration

To examine the relationship between biomarkers and deterioration of motor milestones, all patients were categorized into three groups based on their status of motor milestone deterioration. The “persistent” group comprised patients who exhibited certain motor milestones and did not experience deterioration by the conclusion of the follow-up period. The “lost” group comprised patients who had previously acquired certain motor milestones but had lost them by the end of the follow-up period. In addition, patients who exhibited no specific motor function were included in the “never” group. Table 7 illustrates the distributions of SMN2 and NAIP copy numbers across the three groups, whereas Table 8 depicts the levels of fl-SMN2 and PLS3 expression in relation to the deterioration of the five motor milestones. The results demonstrated a negative correlation between the number of SMN2 copies, NAIP copies, and the level of fl-SMN2 transcript expression, and the deterioration of motor milestones, including head control, rolling, sitting, standing, and walking. Nevertheless, a correlation between motor milestone deterioration and PLS3 expression was observed exclusively in the context of standing and walking abilities.

**Table 7:** SMN2 and NAIP copy number distribution in different motor milestone deterioration levels.

Motor milestones			Preserved	Loss	Never	Kendall’s tau-c	*P* for Trend
Head control		N	101	6	16		
	SMN2	2	1	2	16	−0.380	**<0.001**
		3	86	4	0		
		4	14	0	0		
	NAIP	0	6	1	8	−0.186	**<0.001**
		1	65	2	8		
		2	30	3	0		
Rolling		N	78	17	28		
	SMN2	2	1	1	17	−0.396	**<0.001**
		3	63	16	11		
		4	14	0	0		
	NAIP	0	5	1	9	−0.248	**<0.001**
		1	45	15	15		
		2	28	1	4		
Sitting		N	84	9	30		
	SMN2	2	0	1	18	−0.398	**<0.001**
		3	70	8	12		
		4	14	0	0		
	NAIP	0	6	0	9	−0.212	**0.001**
		1	50	7	18		
		2	28	2	3		
Standing		N	33	9	81		
	SMN2	2	0	0	19	−0.347	**<0.001**
		3	20	8	62		
		4	13	1	0		
	NAIP	0	1	0	14	−0.248	**<0.001**
		1	17	8	50		
		2	15	1	17		
Walking		N	25	12	86		
	SMN2	2	0	0	19	−0.344	**<0.001**
		3	13	10	67		
		4	12	2	0		
	NAIP	0	1	0	14	−0.183	**0.004**
		1	12	9	54		
		2	12	3	18		

SMN2, SMN2 copy number. NAIP, NAIP copy number. NAIP, neuronal apoptosis inhibitory protein; SMN, survival motor neuron. Bold in the table indicates *p* < 0.05.

**Table 8:** fl-SMN2 and *PLS3* expression in different motor milestone deterioration levels.

Motor milestones		Persistent	Lost	Never obtained	*F*	*P*
Head control	*N*	70	0	11		
	fl-SMN2	353.06 ± 126.67	–	193.41 ± 70.96	16.539	**<0.0001**
	PLS3	1.90 ± 0.88	–	1.79 ± 0.80	0.167	0.693
Rolling	*N*	56	8	17		
	fl-SMN2	370.76 ± 127.78	318.49 ± 58.90	207.71 ± 89.90	25.717	**<0.0001**
	PLS3	1.96 ± 0.93	1.54 ± 0.52	1.79 ± 0.75	0.498	0.483
Sitting	*N*	59	5	17		
	fl-SMN2	367.58 ± 127.61	241.04 ± 76.15	232.31 ± 95.89	16.899	**<0.0001**
	PLS3	1.95 ± 0.90	1.90 ± 0.97	1.64 ± 0.69	1.761	0.188
Standing	*N*	23	8	50		
	fl-SMN2	415.82 ± 132.09	321.72 ± 99.76	294.09 ± 120.54	15.624	**<0.0001**
	PLS3	2.24 ± 1.06	1.95 ± 1.06	1.71 ± 0.68	6.246	**0.015**
Walking	*N*	18	10	53		
	fl-SMN2	431.23 ± 139.14	349.25 ± 101.08	294.10 ± 117.86	17.235	**<0.0001**
	PLS3	2.30 ± 1.05	2.06 ± 1.18	1.71 ± 0.67	6.688	**0.012**

Guardians of 81 patients consented for them to undergo transcript-level analysis, Type 1 (*n* = 17), Type 2 (*n* = 36), and Type 3 (*n* = 28). fl-SMN2 and PLS3 expression is shown as mean ± standard deviation. PLS3, F-actin bundling protein plastin 3; SMN, survival motor neuron. Bold in the table indicates *p* < 0.05.

## Discussion

The results demonstrated that disease severity and motor function deterioration exhibited notable differences among SMA types 1–3 and subtypes, particularly among the three subtypes of Type 1. The results of the survival analysis indicated that approximately 50% of the patients with SMA Type 1 reached composite endpoints, which is consistent with the findings reported in the German dataset (19). Furthermore, the endpoint-free survival probability of patients with SMA Type 1 at 10 years was 34.7%, which was comparable to the 30% observed in a previous study conducted in Hong Kong (20). However, this value was slightly higher than the 19.6% reported in a Chinese cohort study conducted 10 years ago (12). This may be attributed to the recent promotion of multidisciplinary therapy in China, which encompasses respiratory intervention and enhancement of guardians and medical care. In patients with SMA Type 1, the mean age of onset and survival status of the three subtypes (types 1a, 1b, and 1c) differed significantly, which is comparable to the findings of previous international studies (9, 19). Our findings indicate that patients with SMA Type 1a exhibited the most severe phenotype and the poorest survival status. The SMA Type 1b phenotype was also relatively severe; however, the composite endpoint survival probabilities before 2 years demonstrated a slight improvement compared to the findings reported by Wijngaarde et al. (9), before subsequently declining significantly to 0% at 4 years. This decline was consistent with the findings reported by Wijngaarde et al. (9) (Table 3). Patients with Type 1c disease exhibited the mildest phenotype, superior achievement of motor function (100% head control and 53.8% rolling), and enhanced survival status, comparable to the findings reported by Wijngaarde et al. (9). These findings confirm the importance of accurately assessing and evaluating the response to DMT treatment by distinguishing between patients with SMA Type 1c and those with SMA types 1a and 1b.

Patients with Type 1 SMA are prone to feeding difficulties and require respiratory support (8, 9). Previous studies have demonstrated that ventilation and gastrostomy tube feeding can effectively improve the survival status of SMA Type 1 and that these interventions are independent influencing factors of survival (19). A multidisciplinary approach to treatment is of paramount importance for the management, prolongation of survival, and improvement of the quality of life of patients with SMA (21, 22). Although 11 patients (36.7%) exhibited feeding difficulties in our study, gastrostomy tube feeding was not performed. A similar phenomenon was observed in another Chinese SMA study (23). Moreover, only three patients with SMA Type 1 in our study used respiratory support, with both interventions being employed at a markedly lower rate than that reported in European studies (8, 19). The acceptance of gastrostomy surgery among parents of children with SMA in China was markedly low, potentially due to cultural differences and the traumatic nature of surgery. Therefore, further efforts must be made to promote education and the implementation of effective digestive and nutritional interventions to improve the survival status of patients with SMA Type 1. In addition, scoliosis is a prevalent complication of SMA, predominantly affecting patients with SMA 1c and 2. However, only a few patients in our cohort underwent surgery for scoliosis. As reported by Wijngaarde et al. (24), the lifetime probability of scoliosis surgery is as high as 80% for SMA types 1c and 2. This may be attributed to the fact that multidisciplinary management remains a relatively novel concept in China and has not yet gained widespread acceptance. It is therefore imperative to disseminate information regarding the aforementioned method and to educate guardians to strengthen the multidisciplinary management of Chinese patients with SMA. This should include the observation and intervention of breathing and feeding in SMA Type 1 and management of scoliosis in types 1c and 2. This could further enhance the quality of life of Chinese patients with SMA and ensure optimal utilization of benefits from SMA DMT.

A negative correlation was observed between the SMN2 copy number and disease severity, which is consistent with the results of previous studies (5, 13, 25, 26). Prior research has indicated a correlation between SMN2 copy number and the survival status of patients with SMA Type 1 (27). The results of the multivariate analysis in the current study align with these findings. Furthermore, a negative correlation was observed between the number of SMN2 copies and the severity of the three subtypes of Type 1. The presence of three copies was observed exclusively in the 1c cases, which manifested at a later age and exhibited enhanced motor development, including head control and rolling. Furthermore, the survival conditions of these children were markedly improved, which is consistent with the findings of Wadman et al. (8) and Wijngaarde et al. (9). In addition, our results indicate that SMN2 copies exerter a pronounced influence on the acquisition and deterioration of motor milestones. Considering these findings, it can be posited that SMN2 copy number may serve as a robust biomarker for the assessment of SMA severity, progression, and prognosis, particularly in the context of SMA Type 1 subtypes.

The NAIP is situated within the same chromosome region (5q13) as SMN1 and SMN2 (4). Several studies have demonstrated that when the range of SMN1 gene deletions encompasses the NAIP gene, patients exhibit a more severe phenotype (28, 29). The NAIP copy number distribution observed in patients with SMA types 1–3 in the current study was consistent with that reported in previous studies conducted in South Korea (30) and Argentina (31). Furthermore, our data indicated that the NAIP copy number was associated with disease severity, motor milestone deterioration, and survival. The median survival period of patients with no copies was significantly shorter than that of patients with two copies. The P44 (GTF2H2) gene has been linked to large-scale deletions associated with a severe form of SMA (32). In addition, H4F5 is frequently deleted in Type I SMA, making it a potential phenotypic modifier of SMA (33). He et al. (34) reported that seven patients who carried a large-scale deletion, including SMN1, NAIP, and GTF2H2, developed severe SMA. Liu et al. (35) observed that approximately 4% of the patients with SMA they examined exhibited a homologous deletion on GTF2H2. Given the research, it has become evident that the absence of NAIP, GTF2H2, or H4F5 is significantly correlated with the severe form of SMA. In future studies, we will consider including these factors in our evaluation.

Our findings revealed that the combined genotype 0–3-1 was the most prevalent and was observed across types 1c–3, as previously documented in other studies (13, 36, 37). In addition, the minimum copy number combined genotypes 0–2-0 and 0–2-1 were exclusively observed in the most severe Type 1a or 1b cases and were associated with decreased survival. Thus, our study corroborates the findings of previous research, indicating that SMN2 and NAIP exert a synergistic effect on the SMA phenotype in a Chinese population (4, 5, 8, 13). Thus, it can be concluded that as the copy number increases, the age of onset is delayed and the mortality rate is reduced. Consequently, NAIP copy number is a significant biomarker that can be used to evaluate the phenotype and survival of SMA. Furthermore, a comprehensive analysis must be conducted in conjunction with SMN1 and SMN2 copy numbers within the Chinese healthcare system.

The transcript level of fl-SMN2 was included in the biomarker column for analysis. Crawford et al. (38) reported that “fl-SMN2 is related to the SMA phenotype, and SMN2 copy number is related to the SMA phenotype.” However, no correlation was found between the SMN2 copy number and fl-SMN2 expression levels. Nevertheless, our study demonstrated a correlation between fl-SMN2 expression and SMN2 copy number (*p <* 0.001). Perhaps the disparate distribution of SMN2 copy numbers may cause this inconsistency. In a study conducted by Crawford et al. (38), the distribution of SMN2 copy numbers was dispersed, with values ranging from 2 to 6. In addition, five or six copies were observed in both Type 1 and Type 2 patients. The SMN2 copy number observed in our study ranged from 2 to 4. Two copies were predominantly identified in patients with Type 1 SMA, whereas four copies were exclusively present in patients with Type 3 SMA. This study revealed that the fl-SMN2 transcript level in patients with SMA Type 2b was markedly elevated compared to that observed in Type 2a, whereas no discernible difference was noted in the SMN2 and NAIP copies between the various subtypes. The fl-SMN2 transcript level may be influenced by several factors in patients with equal copy numbers but differing fl-SMN2 transcript levels (39). First, alternative splice-modifying variants in SMN2, such as c.859G>C and c.835-44G>A, have been demonstrated to promote the full-length transcript containing exon 7 by regulating the splicing of SMN2 (4, 40, 41). Second, methylation levels have been demonstrated to influence the activity of the SMN2 promoter on transcripts (42, 43). Third, partial deletion of the SMN2 gene has been shown to influence the levels of full-length SMN2 transcripts (44, 45). Consequently, the level of fl-SMN2 transcripts may serve as a more precise indicator of the actual expression of the SMN protein than the SMN2 copy number. Moreover, it serves as a robust biomarker for assessing phenotype, progression, motor milestone deterioration, and the outcome of SMA Type 1–3, demonstrating particular efficacy in evaluating phenotypic differences in subtypes of Type 2.

PLS3, which is located on the X chromosome, has been shown to reduce disease severity when its expression is upregulated in female patients. Similarly, PLS3 was identified as a protective modifier of the SMA phenotype (46, 47). The upregulation of PLS3 expression has been observed to alleviate disease severity (4). Cao et al. (15) and Oprea et al. (46) demonstrated that PLS3 is a protective factor in women, with high expression levels observed in females aged >3 years. Our findings indicate a correlation between PLS3 expression and the severity of Type 1–3 phenotypes (48). Despite no statistically significant differences in age and sex between the study groups, our data revealed a tendency for higher PLS3 expression in female patients than in their male counterparts. Furthermore, PLS3 expression was higher in patients with SMA Type 3b than in those with SMA Type 3a. Moreover, there is a correlation between PLS3 and deterioration of motor milestones, specifically the ability to stand and walk, in older patients. However, the correlation between PLS3 expression and disease severity is not absolute. Other disease-modifying genes (X-linked USP9X, UBA, and PLS1) may also affect disease phenotype. It may be advisable to expand the sample size or incorporate additional modifier genes to further assess the efficacy of PLS3 as a phenotypic biomarker in future studies (49).

Our findings indicated that the progression of motor milestones is accelerated and occurs earlier in patients with more severe phenotypes. The deterioration of head control was the shortest, whereas the deterioration of walking ability was the most prolonged. Furthermore, no significant difference was observed in the age at acquisition of motor milestones among patients with the different subtypes. However, a significant difference was noted in the age at motor milestone deterioration. For instance, in types 1c, 2a, and 2b, rolling was achieved at 4 months of age. However, all these types exhibited a distinctive pattern: the more severe the phenotype, the faster and earlier the deterioration of motor milestones occurred. In a study by Mercuri et al. (50), the deterioration of motor milestones in patients with SMA Type 2 was non-linear. There was an overall improvement trend before 5 years of age, which then degenerated rapidly until it became relatively stable after puberty (14 years of age). However, due to the lack of long-term follow-up, our study only observed eight patients with SMA Type 2a and one patient with SMA Type 2b, who exhibited deterioration while sitting. In addition, no apparent age-related boundaries were identified. As the follow-up period is extended to encompass the later stages, it will be possible to ascertain whether the deterioration in sitting exhibits non-linear progression. Furthermore, our findings indicated that the SMN2 and NAIP copy numbers and the fl-SMN2 transcript level were significantly correlated with the five motor milestones, exerting a notable influence on both the acquisition and deterioration of these milestones. In contrast, PLS3 transcript levels demonstrated a correlation solely with the acquisition and deterioration of standing and walking.

This study had several limitations. First, the cohort sample size was insufficient, particularly regarding the limited number of patients with SMA 1a and 3b, and the lack of RNA research data. In addition, the inclusion of data obtained from parental historical reports may have introduced bias into the results. Second, there was a dearth of long-term follow-up data, and no deaths among patients with types 2 and 3 were observed. In addition, no evident boundaries of deterioration according to age were identified. As SMA enters the era of DMT, there is an increasing number of patients receiving DMT treatment. Consequently, extending the follow-up period to obtain more valuable natural history data in DMT-naïve patients is a significant future challenge. Third, the dataset lacked information on motor scale assessments, which would have enabled researchers to assess the patients’ daily motor function and level of impairment. Finally, insufficient blood samples and instability of the extracted SMN protein precluded the use of SMN protein testing. Further research is required to elucidate the relationship between SMN2 copy number, SMN2 transcript level, SMN protein level, and severity of clinical problems.

In conclusion, this study demonstrated the clinical characteristics of DMT-naïve Chinese patients with SMA subtypes 1–3. Our findings indicate that SMN2 and NAIP copies, fl-SMN2, and PLS3 transcript levels were associated with the SMA phenotype and motor milestone deterioration. Among these factors, SMN2 copy number demonstrated a pronounced impact on endpoint survival analysis. In addition, fl-SMN2 expression may serve as the most direct biomarker for disease severity and motor milestone deterioration progression in patients with SMA Type 2 (Type 2a and Type 2b). The data presented provide a valuable baseline for future studies and may be used as a reference point for the comparison of treatment outcomes, prognosis, and follow-up procedures in patients with SMA. To enhance the reliability of the findings, future studies should incorporate a larger sample size and longer follow-up period.

## Data availability statement

The data presented in the study are deposited in the https://www.ncbi.nlm.nih.gov/clinvar/, accession number SCV005200359.

## Ethics statement

The studies involving humans were approved by Ethics Committee of the Capital Institute of Pediatrics (approval no.: SHERLL2017007). The studies were conducted in accordance with the local legislation and institutional requirements. Written informed consent for participation in this study was provided by the participants’ legal guardians/next of kin.

## Author contributions

SO: Conceptualization, Data curation, Formal analysis, Investigation, Validation, Writing – original draft, Writing – review & editing. XP: Conceptualization, Investigation, Validation, Writing – review & editing. WH: Data curation, Writing – original draft. JB: Data curation, Conceptualization, Writing – original draft. HW: Data curation, Writing – review & editing. YJ: Data curation, Writing – review & editing. HJ: Data curation, Writing – review & editing. MW: Formal analysis, Writing – original draft, Conceptualization. XG: Funding acquisition, Data curation, Writing – review & editing. FS: Funding acquisition, Resources, Methodology, Project administration, Supervision, Writing – review & editing, Investigation. YQ: Methodology, Project administration, Resources, Supervision, Conceptualization, Data curation, Formal analysis, Funding acquisition, Investigation, Validation, Visualization, Writing – review & editing.
