# Corrigendum: Association among biomarkers, phenotypes, and motor milestones in Chinese patients with 5q spinal muscular atrophy types 1–3

**DOI:** 10.3389/fneur.2024.1512459

**Published:** 2024-10-29

**Authors:** Shijia Ouyang, Xiaoyin Peng, Wenchen Huang, Jinli Bai, Hong Wang, Yuwei Jin, Hui Jiao, Maoti Wei, Xiushan Ge, Fang Song, Yujin Qu

**Affiliations:** ^1^Department of Medical Genetics, Capital Institute of Pediatrics, Beijing, China; ^2^Department of Neurology, Children's Hospital Affiliated to Capital Institute Pediatrics, Beijing, China; ^3^Center of Clinical Epidemiology, TEDA International Cardiovascular Hospital, Tianjin, China

**Keywords:** spinal muscular atrophy, biomarkers, severity, motor milestones, survival

In the published article, the reference for (14) was incorrectly written as: Zerres K. Natural history in proximal spinal muscular atrophy. Arch Neurol. (1995) 52:518–23. doi: 10.1001/archneur.1995.00540290108025.

It should be: Zerres K, Rudnik-Schöneborn S. Natural history in proximal spinal muscular atrophy. Clinical analysis of 445 patients and suggestions for a modification of existing classifications. *Arch Neurol*. (1995) 52:518–23. doi: 10.1001/archneur.1995.00540290108025.

The reference for (28) was incorrectly written as: Anderson K, Talbot K. Spinal muscular atrophies reveal motor neuron vulnerability to defects in ribonucleoprotein handling. Curr Opin Neurol. (2003) 16:595–9. doi: 10.1097/00019052-200310000-00005.

It should be: Anderson K, Talbot K. Spinal muscular atrophies reveal motor neuron vulnerability to defects in ribonucleoprotein handling. *Curr Opin Neurol*. (2003) 16:595–9. doi: 10.1097/01.wco.0000093102.34793.13.

The reference for (45) was incorrectly written as: Mazoyer S, Vijzelaar R, Snetselaar R, Clausen M, Mason AG, Rinsma M, et al. The frequency of SMN gene variants lacking exon 7 and 8 is highly population dependent. PLoS One. (2019) 14:e0220211. doi: 10.1371/journal.pone.0220211.

It should be: Vijzelaar R, Snetselaar R, Clausen M, Mason AG, Rinsma M, Zegers M, et al. The frequency of SMN gene variants lacking exon 7 and 8 is highly population dependent. *PLoS One*. (2019) 14:e0220211. doi: 10.1371/journal.pone.0220211.

The authors apologize for this error and state that this does not change the scientific conclusions of the article in any way. The original article has been updated.

