# Supplementary material for: Association among biomarkers, phenotypes, and motor milestones in Chinese patients with 5q spinal muscular atrophy types 1–3
Source: Front Neurol. 2024 Sep 2;15:1382410. doi: 10.3389/fneur.2024.1382410 (PMC11404040; doi:10.3389/fneur.2024.1382410)
Supplement: Supplementary file 1 [file Table_1.DOCX]

**Supplementary Information**

**Table S Clinical characteristics and biomarker data of SMA type 1-3**

|  |  | Type 1 (n=30) | Type 2 (n=56) | Type 3 (n=37) | Statistic | P value |
| --- | --- | --- | --- | --- | --- | --- |
| Sex (M:F) | | 11:19 | 29:27 | 19:18 | χ^2^ =2.032 | 0.362 |
| Age, months^ǂ^ | | 17.5 (2–121) | 61 (11–232) | 74 (33–518) | χ^2^ =32.382 | **<0.001** |
| Age at onset, months^ǂ^ | | 3 (0–8) | 10 (4–18) | 21 (10–180) | χ^2^ =91.698 | **<0.001** |
| Death (%) | | 15 (50) | 0 (0) | 0 (0) | Fisher’s exact test | **<0.001** |
| History of feeding difficulties, n (%) | | 11 (36.7) | 5 (8.9) | 0 (0) | Fisher’s exact test | **<0.001** |
| Age at feeding difficulties started, months^ǂ^ | | 7 (2–24) | 22 (11–180) | NA | u=6.249 | **0.012** |
| Respiratory support, n (%) | | 3 (10) | 2 (3.5) | 1 (2.7) | Fisher’s exact test | 0.364 |
| Scoliosis, n (%) | | 8 (26.7) | 21 (37.5) | 8 (21.6) | χ^2^=2.144 | 0.342 |
| Age at diagnosis of scoliosis, months^ǂ^ | | 16 (6–60) | 36 (8–108) | 108 (28–288) | χ^2^=11.762 | **<0.001** |
| Motor milestones, n (%) | |  |  |  |  |  |
|  | Head control | 14 (46.7) | 56 (100) | 37 (100) |  |  |
|  | Rolling | 7 (23.3) | 51 (91.1) | 37 (100) |  |  |
|  | Sitting | 0 (0) | 100 (100) | 100 (100) |  |  |
|  | Standing | 0 (0) | 5 (8.9) | 37 (100) |  |  |
|  | Walking | 0 (0) | 0 (0) | 100 (100) |  |  |
| SMN2 copy number, n | |  |  |  |  |  |
|  | 2 | 18 | 1 | 0 | −0.519 | **<0.001^*^** |
|  | 3 | 12 | 55 | 23 |  |  |
|  | 4 | 0 | 0 | 14 |  |  |
| NAIP copy number, n | |  |  |  |  |  |
|  | 0 | 9 | 5 | 1 | −0.274 | **<0.001^*^** |
|  | 1 | 18 | 36 | 21 |  |  |
|  | 2 | 3 | 15 | 15 |  |  |
| Genotype | |  |  |  | −0.475 | **<0.001^*^** |
|  | 0-2-0 | 9 | 0 | 0 |  |  |
|  | 0-2-1 | 9 | 0 | 0 |  |  |
|  | 0-2-2 | 0 | 1 | 0 |  |  |
|  | 0-3-0 | 0 | 5 | 1 |  |  |
|  | 0-3-1 | 9 | 36 | 20 |  |  |
|  | 0-3-2 | 3 | 14 | 2 |  |  |
|  | 0-4-1 | 0 | 0 | 1 |  |  |
|  | 0-4-2 | 0 | 0 | 13 |  |  |
| fl-SMN2 expression^§^ | | 232.31 ± 95.89 | 323.28 ± 117.07 | 401.95 ± 131.13 | 21.762 | **<0.001^*^** |
| PLS3 expression^§^ | |  |  |  |  |  |
|  | Total | 1.64 ± 0.69 | 1.74 ± 0.66 | 2.21 ± 1.09 | 5.018 | **0.028^*^** |
|  | Male | 1.50 ± 0.51 | 1.58 ± 0.62 | 2.14 ± 0.96 | 2.697 | 0.081^*^ |
|  | Female | 1.71 ± 0.78 | 1.90 ± 0.68 | 2.30 ± 1.25 | 1.344 | 0.273^*^ |

^*^P for trend

^ǂ^ Median (minimum-maximum)

^§^ Mean ± standard deviation

F, female; M, male; NA, not applicable; SMN, survival motor neuron

Guardians of 81 patients consented for them to undergo transcript-level analysis, type 1 (n=17), type 2 (n=36), and type 3 (n=28)


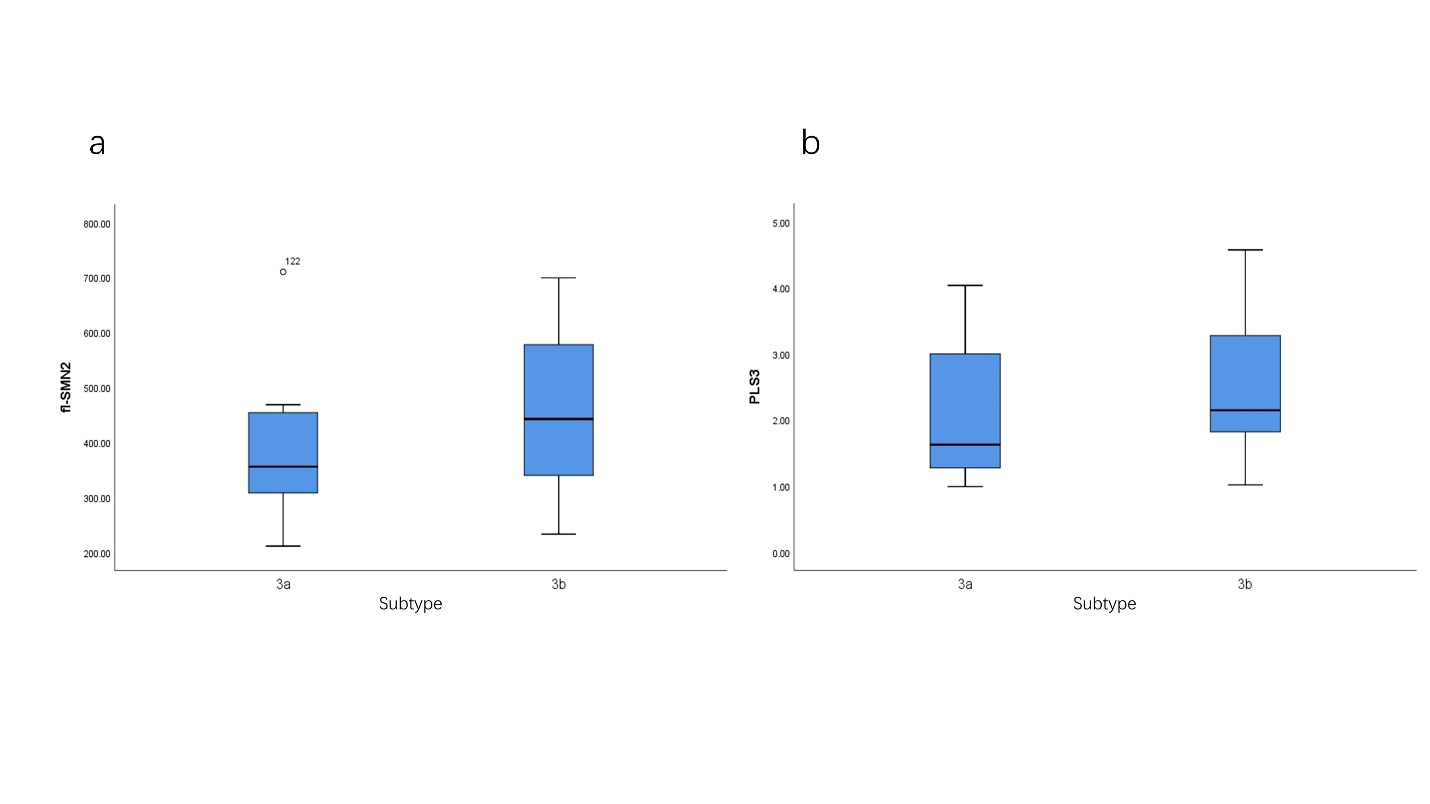


**Figure S** Expression of fl-SMN2 and PLS3 in SMA type 3a and 3b**.** **a** Expression of fl-SMN2 in SMA type 3a and 3b. **b** Expression of PLS3 in SMA type 3a and 3b. Guardians of 81 patients consented for them to undergo transcript-level analysis.
